# Comprehensive Analysis of Hepatitis Delta Virus Assembly Determinants According to Genotypes: Lessons From a Study of 526 Hepatitis Delta Virus Clinical Strains

**DOI:** 10.3389/fmicb.2021.751531

**Published:** 2021-11-16

**Authors:** Athenaïs Gerber, Frédéric Le Gal, Samira Dziri, Chakib Alloui, Dominique Roulot, Paul Dény, Camille Sureau, Ségolène Brichler, Emmanuel Gordien

**Affiliations:** ^1^Laboratoire de Microbiologie Clinique, Université Paris Nord, Sorbonne Paris Cité, Hôpitaux Universitaires de Paris-Seine-Saint-Denis, Bobigny, France; ^2^Centre National de Référence des Hépatites B, C et Delta, Hôpitaux Universitaires de Paris-Seine-Saint-Denis, Bobigny, France; ^3^INSERM U955, Équipe 18, Institut Mondor de Recherche Biomédicale, Créteil, France; ^4^Unité d’Hépatologie, Université Paris Nord, Sorbonne Paris Cité, Hôpitaux Universitaires de Paris-Seine-Saint-Denis, Bobigny, France; ^5^Inserm, U1052 – UMR CNRS 5286, Centre de Recherche en Cancérologie de Lyon, Lyon, France; ^6^Laboratoire de Virologie Moléculaire, Institut National de la Transfusion Sanguine, Paris, France

**Keywords:** HDV, editing, genotype, HDAg, next-generation-sequencing, pathogenesis

## Abstract

Human hepatitis Delta virus (HDV) infection is associated to the most severe viral hepatic disease, including severe acute liver decompensation and progression to cirrhosis, and hepatocellular carcinoma. HDV is a satellite of hepatitis B virus (HBV) that requires the HBV envelope proteins for assembly of HDV virions. HDV and HBV exhibit a large genetic diversity that extends, respectively to eight (HDV-1 to -8) and to ten (HBV/A to/J) genotypes. Molecular determinants of HDV virion assembly consist of a C-terminal Proline-rich domain in the large Hepatitis Delta Antigen (HDAg) protein, also known as the Delta packaging domain (DPD) and of a Tryptophan-rich domain, the HDV matrix domain (HMD) in the C-terminal region of the HBV envelope proteins. In this study, we performed a systematic genotyping of HBV and HDV in a cohort 1,590 HDV-RNA-positive serum samples collected between 2001 to 2014, from patients originated from diverse parts of the world, thus reflecting a large genetic diversity. Among these samples, 526 HBV (HBV/A, B, C, D, E, and G) and HDV (HDV-1, 2, 3, and 5 to -8) genotype couples could be obtained. We provide results of a comprehensive analysis of the amino-acid sequence conservation within the HMD and structural and functional features of the DPD that may account for the yet optimal interactions between HDV and its helper HBV.

## Introduction

The human Hepatitis Delta virus (HDV) is a satellite of hepatitis B virus (HBV), recruits the HBV envelope proteins for HDV virion assembly and, hence, propagation. In 2019, 296 million individuals were thought to be chronically infected with HBV worldwide (WHO, Global progress report on HIV, viral hepatitis and sexually transmitted infections, 2021, page 8) and up to 60 millions of them are coinfected with HDV (Chen et al., 2019; Stockdale et al., 2020).

Hepatitis delta virus is responsible for the most severe viral hepatitis, and chronically infected individuals may progress to liver fibrosis, cirrhosis decompensation and hepatocellular carcinoma (HCC). In a recent retrospective study on 1,112 HDV-infected patients, 48.8% of the patients had developed cirrhosis, 24.2% had one or more episodes of hepatic decompensation (HD), and 9.2% had hepatocellular carcinoma (HCC) (Roulot et al., 2020) during the follow-up period.

**FIGURE 1:**
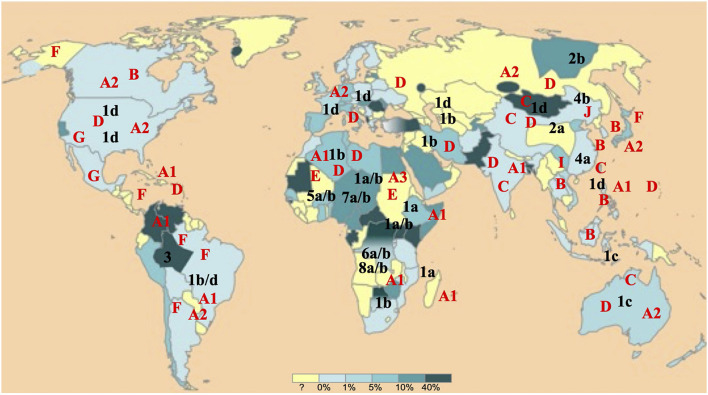
Worldwide hepatitis B virus (HBV) and Hepatitis Delta Virus (HDV) (sub)genotypes distribution. HBV and HDV (sub)genotypes are denoted, respectively in red and in black. The world map is colored according to a gradient of total anti-HDV antibody prevalence to up to 40% drawn from almost all most international publications (adapted from EMC – Biologie médicale 2018;13(3):1–9).

Hepatitis Delta Virus and Hepatitis B Virus segregate, respectively into 8 (HDV-1 to -8) and 10 (HBV/A to/I, and a putative J) genotypes and in several subgenotypes within genotypes which share a characteristic geographical distribution (Figure 1; Pourkarim et al., 2014; Le Gal et al., 2017a).

For HDV virion assembly, the HDV ribonucleoprotein (RNP) composed of HDV RNA bound to multiple copies of the small (S) and large (L) isoforms of HDV antigen (HDAg) proteins (Ryu et al., 1992, 1993; Lin et al., 2010), must interact with the HBV envelope proteins. The determinants of HDV assembly on the RNP consists of a proline-rich domain (PRD) at the C-terminal region of L-HDAg named the Delta packaging domain (DPD) (Jenna and Sureau, 1998, 1999; Blanchet and Sureau, 2006; Komla-Soukha and Sureau, 2006; Dastgerdi et al., 2012; Shirvani-Dastgerdi and Tacke, 2015) that includes a farnesylation signal that is crucial to virion assembly (Glenn et al., 1992; Bordier et al., 2003; Taylor, 2006). The determinants of HDV assembly in the HBV envelope proteins consists of a tryptophan-rich domain at the C-terminal region of the HBV surface protein (HBs) named the HDV matrix domain (HMD). In this study, we analyzed the conservation of the HDV assembly determinants using a panel of 1,590 HDV-RNA-positive serum samples collected between 2001 to 2014 by the French National Reference Centre for Viral Hepatitis B, C, and D (FNRC-D), according to HBV and HDV genotypes. All samples were from patients diagnosed in France but originating from diverse parts of the world and who have been infected in their country of origin. We performed a systematic HBV and HDV genotyping and carefully analyzed the amino-acid sequences of the HDV assembly determinants.

## Materials and Methods

### Samples

As part of our missions of FNRC-D located at the Avicenne Hospital in Bobigny (expertise, advice, epidemiological surveillance and alerts, described in the Official Journal of the Republic of France, JO n°141 of June 08, 2016, text 10 of 118), we took advantage of our large collection of samples. Samples are those of all newly diagnosed HDV-infected patients in France, by detection of total anti-HDAg antibodies (anti-HDAg) and HDV-RNA in serum or plasma. Anti-HDAg antibodies are detected using the DiaSorin Anti-HDAg ELISA (DiaSorin Inc., Antony, France). HDV-RNA was detected using an in house real-time HDV RT-PCR (Le Gal et al., 2005) or the Eurobioplex HDV RNA kit (Le Gal et al., 2017b) or by a qualitative HDV-RT-PCR. Most of the 1,590 samples considered in this study, were collected in France from 2001 to 2014, but, in order to encompass a large HDV genetic diversity, samples from patients originated from diverse parts of the world, were also included. Finally, samples were originated from nearly all continents and from individuals who had been infected during childhood or adolescence, in their country of birth, and who were mostly naïve of treatments (Figure 1).

### Hepatitis B Virus and Hepatitis Delta Virus Genotyping

Hepatitis B Virus and Hepatitis Delta Virus genotyping were performed using direct sequencing of DNA amplicons of the 3′ end of HBsAg ORF and the 3′ end of the L-HDAg ORF. A nested PCR was systematically performed for amplification of HBV DNA (Williams et al., 2009). For first round PCR, a 795 base-pair (bp)-fragment was amplified using the PolT1 (5′-TCA CAA TAC CRC AGA GTC TAG AC-3′) and the PolT2 primers (5′-GGR GCR GCA AAR CCC AAA AGA CC-3′). For second round, a 410 bp amplicon was obtained using the inner primers Pol-1 (5′-TCA AGG TAT GTT GCC CG-3′) and Pol-2d (5′-TAA CCC CAK CKT TTK GTT TT-3′). For HDV, genotyping was performed as described earlier by direct sequencing of a 400-nucleotide (nt) *R0* region of the genome spanning nt 889 to 1289. The purified amplicons were bidirectionally sequenced by the Sanger method using the automated sequencer 3500Dx (Life technology^®^) followed by extensive phylogenetic analyses as described (Le Gal et al., 2017a).

### Hepatitis B Virus and Hepatitis Delta Virus Protein Sequence Analyses

**FIGURE 2:**
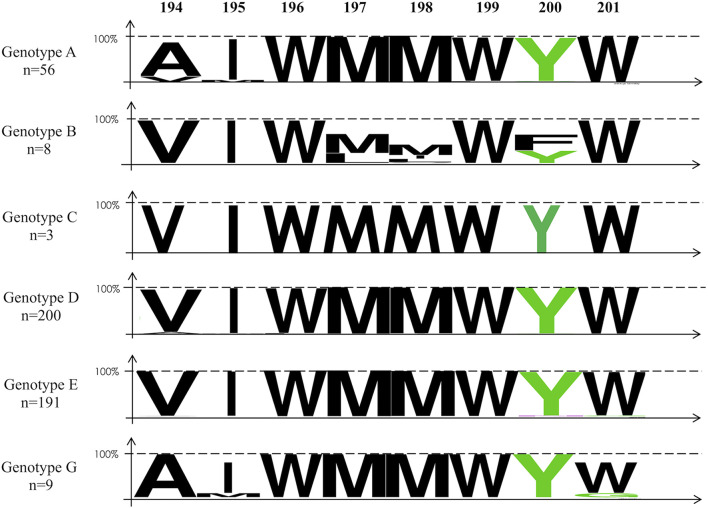
Alignment of the amino-acid sequences of the HDV matrix domain within the cytosolic loop II of the HBV envelope protein using the Web Logo representation according to genotypes. The colors correspond to the physico-chemical properties of the amino acids: polar amino acids G, S, T, Y, and C (green); negatively charged amino acids Q and N (purple); basic amino acids K, R, and H (blue); acidic amino acids D and E (red); and hydrophobic amino acids A, V, L, I, P, W, F, and M (black). On the top of the figure: Amino-acid position. On the left, the number (n) of sequences analyzed for each genotype group. Accession numbers of the sequence of the strains used: AY862864; AB033558; AB033559; AB036920; AB048701; AB059661; AB076678; AB086397; AB104712; AB106564; AB116549; AB194951; AB194952; AB205127; AB222713; AB246335; AB274976; AB298362; AB453987; AB493845; AB493848; ABO56513; ABO56514; AF090839; AF090841; AF090842; AF223965; AF297624; AJ309371; AJ627219; AJ627221; AM180623; AM184125; AM494691; AY090455; AY090461; AY161140; AY179734; AY233288; AY236164; AY311369; AY796031; AY934767; D00329; D00331; D00630; D12980; D16665; D23677; DQ315776; DQ315779; DQ336679; EF103278; EU239220; EU366129; FJ692532; FJ692554; FJ692556; FJ692561; FJ692597; FJ904405; FJ904442; FM199974; FN594769; FN594771; GQ331046; GQ331048; GU456648; X65257; X85254; Z35716; AJ131574; and AM180624.

**FIGURE 3:**
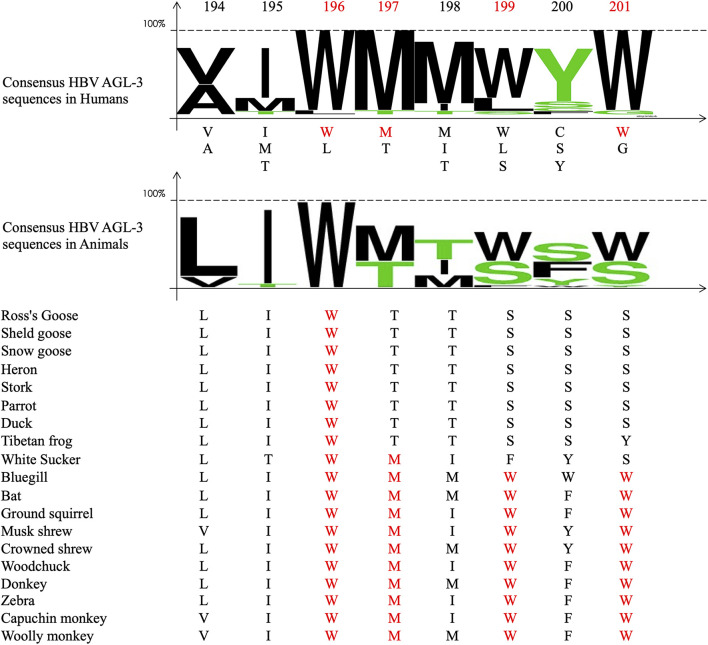
Alignment of the amino-acid sequences of the Delta packaging domain within the C-terminus portion of the large HDV antigen (L-HDAg) protein. On the top of the figure: Amino-acid position. In red the position 196, 197, 199, and 201 of the conserved amino acid in more than 95% of the studied samples (see Table 3). The upper panel. Shows the consensus sequence of the aligned HBV amino acid sequences of the HDV matrix domain of the studied samples in humans. The lower panel shows the Web Logo representation and the sequences of the HDV matrix domain of different HBV animal species retrieved from published reference database. In red the conserved amino acids at the positions of interest. Accession numbers of the sequence of the strains used: AB118841; AB118840; HF679404; GU177114; AJ309879; AJ309880; AM183333; AM183330; AM183327; AM183326; AM183331; AX741154; AX741159; AM183328; AM183332; AJ583887; AM183329; AX741164; AX741149; AJ584847; AX741169; X77627; U81989; X60193; M84917; U81988; U19598; AF018077; D01075; X04451; M58629; L22063; L22061; AB037949; AF309420; AF008373; AF008309; AF008375; AF0008319; AF008374; AF008347; AF104263; AY148020; AM902174; AM902168; AM902164; AM779574; AM902177; AM902180; AM779580; AM902163; AM779575; AM902165; AM902166; AM902167; AM902181; AM902169; AM779578; AM902170; AM902171; AM902172; AM779577; AM902173; AM902179; AM902175; AM902176; and AM779576.

**FIGURE 4:**
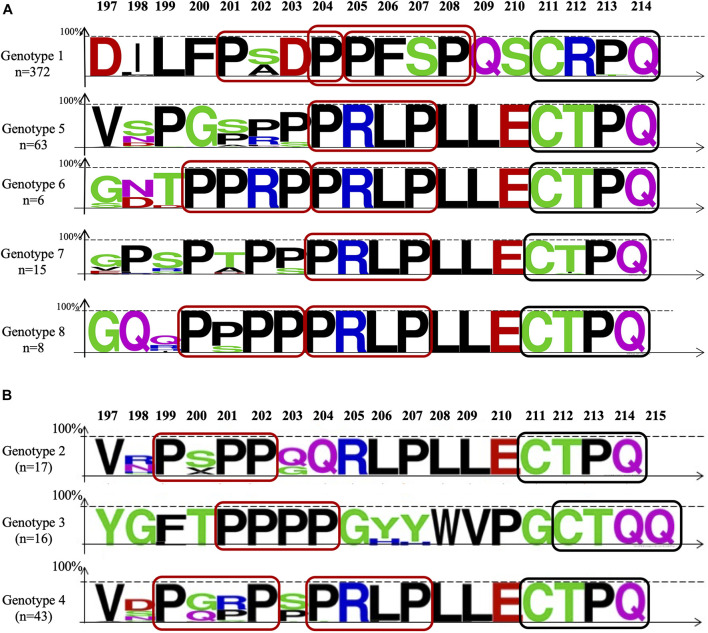
Alignment of the delta packaging domain within the 19 C-terminal amino acids of the large delta protein (L-HDAg) using the Web Logo representation. The colors correspond to the physico-chemical properties of the amino acids: polar amino acids G, S, T, Y, and C (green); negatively charged amino acids Q and N (purple); basic amino acids K, R, and H (blue); acidic amino acids D and E (red); and hydrophobic amino acids A, V, L, I, P, W, F, and M (black). **(A)** The studied samples and **(B)** of HDV-2, -3, and -4 sequences retrieved from international reference database. On the top of the figure: Aa position. On the left, the number (n) of sequences aligned for each genotype group. The red boxes delimitate the PXXP motifs and the PPXXP, and the black ones the farnesylation site.

The deduced amino acid sequences of PRDs and TRDs were analyzed separately for all samples for each genotype. Sequence variability was characterized from genotype-specific multiple alignments with 73 HBV and 67 HDV reference sequences retrieved from GenBank database covering all known HBV and HDV genotypes. The Web Logo application^1^ was used for sequence alignment (Figures 2–4). For HBV alignment, we also included non-human HBV sequences from different species retrieved from reference databases (see Figure 2).

## Results

### Determination of Hepatitis B Virus Genotypes and Hepatitis B Virus/Hepatitis Delta Virus Genotype Combinations

Of the 1590 HDV positive samples of our collection, only 526 HBV amplicons (33.1%) could be obtained using a sensitive nested-PCR. The low efficiency of HBV DNA amplification is likely due to the inhibition of HBV replication exerted by coinfecting HDV (Williams et al., 2009).

Six HBV genotypes were identified: HBV/A (11.8%), HBV/B (1.7%), HBV/C (0.6%), HBV/D (42%), HBV/E (42.2%), and HBV/G (1.7%), from different regions of the world, most of them (50.8%) being from sub-Saharan Africa (Table 1). They were associated with seven HDV genotypes HDV-1 (78.9%), HDV-2 (0.4%), HDV-3 (0.2%), HDV-5 (13.7%), HDV-6 (1.3%), HDV-7 (4%), and HDV-8 (1.5%). Most of the HBV-HDV genotype’ combinations agreed with the already described geographical distribution (Table 2). However, some unexpected associations were observed, such as: a European HBV/A2-genotype associated to an African HDV-8 genotype in a Gabonese patient; an Asian HBV/C with an African HDV-5 in a Sierra-Leonean patient; and an HBV/G with an African HDV-7 in a Cameroonian patient. Similarly, HDV genotypes, such as African HDV-1 and HDV-5 to -8 originated from Africa, are found in European Eastern or Western countries, and conversely European HDV-1 genotypes are observed in African patients, reflected migrations of populations (see Table 2).

**TABLE 1:** Number of HBV genotypes obtained among the 1,590 clinical strains according to the geographic origin of the patients.

World area	Number of sequences	
	
	*N* = 526	(%)
Northern Africa	20	3.8
Sub Saharan Africa	267	50.8
Asia	36	6.8
Western Europe	88	16.7
Eastern Europe	90	17.1
Near and Middle East	24	4.6
South America	1	0.019

**TABLE 2:** HBV and HDV genotypes ‘combinations in the cohort of 526 out of 1,590 patients of French National Reference Centre for hepatitis Delta virus database.

Genotypes	HDV	HDV-1	HDV-2	HDV-3	HDV-5	HDV-6	HDV-7	HDV-8
HBV	N (%)	415 (78.9)	2 (0.4)	1 (0.2)	72 (13.7)	7 (1.3)	21 (4)	8 (1.5)
HBV/A	62 (11.8)	53		1	1		4	3
HBV/B	9 (1.7)	7	2					
HBV/C	3 (0.6)	2			1			
HBV/D	221 (42)	216			5			
HBV/E	222 (42.2)	129			65	7	16	5
HBV/G	9 (1.7)	8					1	

*Number and percentage of strains are indicated.*

### Hepatitis Delta Virus Matrix Domain Amino Acid Sequence Diversity

As shown in Figure 2 and Table 3, the HDM amino acid sequences of 464 out of 526 samples, were aligned with 73 reference sequences retrieved from international databases. Of note, within the sequence of interest 194-VIWMMWYW-201, the amino acids 196W, 197M, 198M, W199, and the 201W were conserved in 99% of all the sequences and the 200Y in more than 98%. Overall, the 196-WMMWYW-201 motif is conserved in 99% of the analyzed sequences (Figure 2 and Table 3).

**TABLE 3:** Amino-acid sequence of the HDV matrix domain within the cytosolic loop II of the HBV envelope at position 194 to 201 of each studied sample (*N* = 502).

Number of samples		HDV Matrix Domain (amino-acid sequence 194–201)							
	
N	(%)	194	195	196	197	198	199	200	201
400	(79.7)	V	I	W	M	M	W	Y	W
65	(12.9)	A	I	W	M	M	W	Y	W
1	(0.2)	A	I	W	M	M	W	C	W
1	(0.2)	A	I	W	M	M	W	S	W
1	(0.2)	V	I	W	T	M	W	Y	W
4	(0.8)	V	I	L	M	M	W	Y	W
5	(0.01)	V	I	W	M	M	W	Y	G
4	(0.8)	V	I	W	M	I	W	Y	W
1	(0.2)	V	I	W	M	T	W	Y	W
3	(0.6)	V	I	W	M	M	W	F	W
4	(0.8)	V	I	W	M	M	W	S	W
2	(0.4)	V	M	W	M	M	W	Y	W
1	(0.2)	V	I	W	M	M	S	Y	W
1	(0.2)	A	I	W	M	M	L	Y	W
1	(0.2)	V	I	W	M	M	L	Y	W
1	(0.2)	A	M	W	M	M	L	Y	W
1	(0.2)	A	M	W	M	I	L	Y	W
5	(0.01)	A	M	W	M	M	W	Y	W
1	(0.2)	A	T	W	M	M	W	Y	W

502		**Percentage of amino acid at different positions**							
		
		**V**	**I**	**W**	**M**	**M**	**W**	**Y**	**W**

		84.8%	98%	99%	99%	98.8%	99%	98.2%	99%

*The percentage of amino acid at each position is indicated.*

Regarding TRD according to genotype, HBV/B appears to be more divergent than other genotypes, with, however, the highly conserved tryptophane residues at position 196, 199, and 201. Similarly, the tyrosine 200 was also conserved in half of the eight available sequences (Figure 2). In non-human HBV sequences, 196W, 197M, 199W, and 201W appear strictly conserved in bats, ground-squirrels, shrews, woodchucks, donkeys, zebras, woolly monkeys, capuchin monkeys, and also in Bluegill sunfish hepatitis B virus sequences (Figure 3; de Carvalho Dominguez Souza et al., 2018; Rasche et al., 2019, 2021). However, TRD is not conserved in avihepdnaviruses sequences in which only 196W is conserved because of the pol gene catalytic domain overlap (Komla-Soukha and Sureau, 2006).

### Delta Packaging Domain Amino Acid Sequence Diversity

Figure 4 panel A shows the amino acid sequence diversity of 464 available sequences of the DPD (the 19-20 C-Terminus domain of the L-HDAg) of HDV strains (371 HDV-1, 63 HDV-5, 6 HDV-6, 175 HDV-7, and 8 HDV-8), aligned with 67 HDV referenced sequences retrieved from international databases. DPD, known to be crucial for HDV virion assembly, is characterized by the abundance of proline residues and/or hydrophobic amino acids situated just upstream the well-described 211-CXXQ-214 farnesylation motif (Figure 4). This latter is involved in the anchorage of the farnesylated RNP to the endoplasmic reticulum lipid membrane in proximity of the envelope proteins. Very interestingly, within this DPD, one or two copies of the well-characterized proline-rich motif PXXP (where X is denotes any amino acid) involved in protein/protein interactions and/or in transduction of cell signaling (Kay et al., 2000; Gao et al., 2006; Kato et al., 2006; Biedermannova et al., 2008) are clearly individualized in all HDV genotypes (Figure 3). Indeed, considering the samples of our study, the 204-PXXP-207 motif is found in all HDV-5 to -8. An additional 200-PXXP-203 motif is also found in genotypes HDV-6 and -8. For HDV-1 which is ubiquitously distributed and associated with most HBV genotypes, two contiguous motifs, 201-PXXP-204 and 205-PXXP-208 are found. Furthermore, overlapping these two PXXP motifs, HDV-1 sequences exhibited at position 204 to 208 (Figure 4A), another proline rich motif yet described, the PPXXP involved in SH3 binding and in the PI3K/Akt signaling pathways activation (Shin et al., 2007).

Considering HDV2, -3, and -4 sequences retrieved from the literature (Figure 4B), HDV-4 exhibited two PXXP motifs, the 204-PXXP-208 described above in HDV-5 -to -8 DPD, and an additional one the 199-PXXP-202. This latter is also found at the same position in HDV-2. As for the HDV-3, the most genetically distant HDV strain, a remarkable 201-PPPP-204 was found in all the 16 HDV-3 HMD.

## Discussion

Hepatitis B virus is a major public health concern affecting more than two-hundred millions of chronically infected individuals worldwide. HDV coinfection considerably worsens the clinical outcome of chronically infected patients, in comparison to HBV mono-infection. HDV is a particularly amazing virus, that absolutely needs provision of HBV surface proteins (HBsAg) to envelop its ribonucleoprotein to constitute its complete viral particle. HDV and HBV are both characterized by a very high genetic diversity in respectively 8 (HDV-1 to -8) and 10 (HBV/A to/J) genotypes, and in several subgenotypes, and by a specific geographical distribution (Figure 1). Thanks to the large collection (1,590 samples) of HDV strains of the FNRC, whose HDV-genotypes had all been characterized, it was particularly tempting to address whether specific or preferential associations between HDV and HBV did exist *in vivo* regardless of the genotypes. Interestingly these strains were isolated from patients originated from most parts of the world (except America and South-East Asia) and who have been infected in their country of birth (Table 1). Because of the availability of this large panel of HDV positive serum samples in our hospital, we investigated the nucleotide sequences of coinfecting HBV and HDV for the conservation of the HMD and DPD amino acid sequences. Because HMD and DPD are key to HBV HDV interaction, it is expected that they display a high level of conservation (Blanchet and Sureau, 2006; Komla-Soukha and Sureau, 2006).

Hepatitis B virus sequences could be obtained from only 33% of the samples due to the suppressive effect of HDV on HBV replication (Williams et al., 2009). Consequently, 526 HBV and HDV genotype couples out of the 1,590 strains considered, could be analyzed. Various combinations between the six HBV and seven HDV genotypes were observed, reflecting their expected geographical distribution (Table 2). The ubiquitous HDV-1 was associated with all HBV genotypes, the Asian HBV (HBV/C) was associated to HDV-2 in Chinese patients. However, unexpected associations were also documented such as the European HBV/A2 with African HDV-8 (Gabon); Asian HBV/C with African HDV-5 (in Sierra Leone); North-African HBV/D7 with European HDV-1 (in Moldavia), and HBV/G with an African HDV-7 (Cameroon). Such associations have been shown to occur in an *in vitro* model between HDV-1 by the envelope proteins of HBV-A to I (Wang et al., 2021). More recently, all known HBV and HDV genotype combinations (HBV/A to H and HDV-1 to -8) were shown to be functional *in vitro* (Freitas et al., 2014). Both studies concluded for genotypic variations for replication competence, envelopment preference, and kinetics of virion secretion. In addition, in this latter *in vitro* model, the most productive combinations did not correlate to the natural expected geographic distribution arguing against an evolutionary adaptation of HDV ribonucleoprotein complex to HBV envelopes.

Thus, it remains to assess by both in *in vivo* and in *in vitro* larger studies whether *in vitro* fitness according to HBV/HDV genotype combination is indicative of potential disease severity. In case of HBV mono-infection genotypes C, D, and F are associated with a higher lifetime risk of cirrhosis and HCC development in comparison to genotypes A and B (Lin and Kao, 2017). HBV-F is often described as responsible for an aggressive course of liver disease, reflected by high histological indexes and high risks of development of hepatocellular carcinoma, and liver-related mortality (Marciano et al., 2013). Interestingly, HDV-3, often associated to HBV/F, is also associated to fulminant hepatitis (Melo Da Silva et al., 2019). However, no comprehensive investigation of clinical significance of HDV genotypes has been performed so far. Ancient studies on small groups and with a limited number of genotypes suggested HDV genotypes might affect the severity of HDV chronic infection. For our part, with the large Deltavir study (Roulot et al., 2020) we showed that European HDV-1 and African HDV-5 were more at risk of developing cirrhosis than in HBV mono-infected patients. HDV, which is known to inhibit HBV replication (Williams et al., 2009; Tham et al., 2020) is therefore mainly involved in the severity of the hepatic disease in chronic HDV infection by mechanisms yet to be fully understood.

We then considered HDV-RNP packaging by the HBsAg at the molecular level. From our analysis, it appears that the amino-acid sequences of the HDV assembly determinants, HMD and DPD are well conserved. The tryptophan-rich motif determinant in HBV envelope proteins 196-WMXWYW-204 is strictly conserved in 99% of the HBV sequences, and even in envelope sequences of hepadnaviruses-infected animals, such as bluegills, bats, woodchucks, ground-squirrels, shrews, donkeys, zebras, capuchin monkeys, and woolly monkeys, but not in Avihepadnaviruses (see Figure 3). Indeed, experimentally, Woodchucks chronically infected with the woodchuck hepatitis virus (WHV) can be superinfected with HDV, leading to WHV/HDV chronic infection, and this species has been used as an animal model for HDV infection (Taylor et al., 1987; Negro et al., 1989). However, it is noteworthy that none of the HBV-infected animals listed above, carried any HDV or an HDV-like virus.

As described in the section “Results,” within the DPD at the C-Terminus of the L-HDAg protein the remarkable motif PXXP (and the PPXXP in HDV-1 stains) was individualized in all HDV strains, in two copies for most of them, therefore providing the molecular and structural bases of HDV RNP envelopment for HDV virion morphogenesis. Indeed, this motif described earlier in several models, is known to be essential for protein/protein interactions with a counterpart tryptophan-rich motif and involved in cell signaling (Sudol et al., 1995; Kay et al., 2000; Zarrinpar and Lim, 2000; Gao et al., 2006; Kato et al., 2006).

However, in a recent study, it was shown that the HDV-RNP could assemble with envelope glycoproteins (Gps) of non-hepadnaviruses such as hepatitis C virus (HCV), vesicular stomatitis virus (VSV), human metapneumovirus, Dengue virus (DENV), and West Nile virus leading to the production of pseudotypes virions that were shown infectious *in vitro* and, in the liver-humanized mouse model. Whether this can occur *in vivo* in the true life, has been shown to date in only one study of Chemin I and collaborators, on 160 Venezuelan patients infected with hepatitis C virus (HCV) (Chemin et al., 2021). Among this cohort, they described one patient negative for all serological and molecular HBV markers, but positive for anti-HDV-Ab and, using a nested RT-PCR, they could also detect HDV-RNA, which could be sequenced and classified within HDV genotype 1 (Chemin et al., 2021).

Since, several *in vivo* studies have been reported on several samples of patients or blood donors infected with different replicative viruses including, HCV, DENV, and Zika virus (Cappy et al., 2021; Pfluger et al., 2021) and Gerber et al. (personal communication and article in preparation). All these studies, while using sensitive methods for HDV RNA detection (Lower limit of detection about 10 IU/mL), failed to find any evidence of concomitant HDV infection in the absence of HBV. Therefore, more *in vivo* studies are needed to assess for confirm or to infirm such hypothesis.

In addition, studies should also be performed at molecular level to identify if any, putative HMDs within the envelope Gps of these viruses. However, according to the worldwide global and relative prevalence of HDV, HCV and other viruses and co-infections, it is reasonable to hypothesize that efficient propagation of HDV infection by alternate helpers than HBV, if this exists, might be very rare and does not represent a public health-care threat.

In summary, all these results raise the main question of the fundamental bases for the helper function of the hepadnaviruses for this amazing HDV. Among them, the unique C-terminal 197-213 amino-acids of the L-HDAg, resulting from an editing process of the HDV antigenome replicative intermediate which cumulates major functional and structural features involved in interactions with the HBsAg. They include a nuclear export signal (NES) between amino acids 197 to 210 *via* a chromosome region maintenance-1 independent pathway (Lee et al., 2001; Huang et al., 2007, 2009, 2013); the farnesylation site, 211-CXPQ-214 (Glenn et al., 1992). Moreover, within the DPD, a clathrin box between amino-acids 199 to 203 has been described. L-HDAg has been proposed as a novel clathrin adaptor-like protein, involved in the post-Golgi membrane trafficking of the farnesylated HDV-RNP (Huang et al., 2007, 2009; Wang et al., 2009), which, interestingly, overlaps the 199-PXXP202 motifs described above involved in TRD/PRD binding. Altogether these remarkable features may account for the yet optimal interactions between HDV and its helper HBV.

## Data Availability Statement

The original contributions presented in the study are included in the article/supplementary material, further inquiries can be directed to the corresponding author.

## Author Contributions

AG, FL, SD, CS, DR, and EG: conceptualization and methodology. AG, CA, PD, FL, SD, SB, and EG: formal analysis, and writing – review and editing. AG and EG: writing – original draft preparation. All authors read and approved the final manuscript.

## Conflict of Interest

The authors declare that the research was conducted in the absence of any commercial or financial relationships that could be construed as a potential conflict of interest. The handling editor declared a shared affiliation with several of the authors FL, CA, DR, SB, PD, and EG at time of review.

## Publisher’s Note

All claims expressed in this article are solely those of the authors and do not necessarily represent those of their affiliated organizations, or those of the publisher, the editors and the reviewers. Any product that may be evaluated in this article, or claim that may be made by its manufacturer, is not guaranteed or endorsed by the publisher.

## Footnotes

1
http://weblogo.berkeley.edu
